# Cytochrome P450 1A1/2, 2B6 and 3A4 HepaRG Cell-Based Biosensors to Monitor Hepatocyte Differentiation, Drug Metabolism and Toxicity

**DOI:** 10.3390/s19102245

**Published:** 2019-05-15

**Authors:** Manuel Vlach, Nicolas Quesnot, Hélène Dubois-Pot-Schneider, Catherine Ribault, Yann Verres, Kilian Petitjean, Claudine Rauch, Fabrice Morel, Marie-Anne Robin, Anne Corlu, Pascal Loyer

**Affiliations:** 1 Inserm, INRA, Univ Rennes, Institut NUMECAN (Nutrition Metabolisms and Cancer) UMR-A 1341, UMR-S 1241, Plateforme BiogenOuest SynNanoVect, F-35000 Rennes, France; manuel.vlach@univ-rennes1.fr (M.V.); quesnotnicolas@hotmail.fr (N.Q.); catherine.ribault@univ-rennes1.fr (C.R.); yann.verres@etudiant.univ-rennes1.fr (Y.V.); petitjeankilian@gmail.com (K.P.); claudine.rauch@univ-rennes1.fr (C.R.); anne.corlu@univ-rennes1.fr (A.C.); 2 Université de Lorraine, CNRS, CRAN, F-54000 Nancy, France; helene.dubois-pot-schneider@univ-lorraine.fr

**Keywords:** HepaRG cells, cell-based biosensors, cytochromes P450, drug screening, hepatotoxicity

## Abstract

Human hepatoma HepaRG cells express most drug metabolizing enzymes and constitute a pertinent in vitro alternative cell system to primary cultures of human hepatocytes in order to determine drug metabolism and evaluate the toxicity of xenobiotics. In this work, we established novel transgenic HepaRG cells transduced with lentiviruses encoding the reporter green fluorescent protein (GFP) transcriptionally regulated by promoter sequences of cytochromes P450 (CYP) 1A1/2, 2B6 and 3A4 genes. Here, we demonstrated that GFP-biosensor transgenes shared similar expression patterns with the corresponding endogenous CYP genes during proliferation and differentiation in HepaRG cells. Interestingly, differentiated hepatocyte-like HepaRG cells expressed GFP at higher levels than cholangiocyte-like cells. Despite weaker inductions of GFP expression compared to the strong increases in mRNA levels of endogenous genes, we also demonstrated that the biosensor transgenes were induced by prototypical drug inducers benzo(a)pyrene and phenobarbital. In addition, we used the differentiated biosensor HepaRG cells to evidence that pesticide mancozeb triggered selective cytotoxicity of hepatocyte-like cells. Our data demonstrate that these new biosensor HepaRG cells have potential applications in the field of chemicals safety evaluation and the assessment of drug hepatotoxicity.

## 1. Introduction

The liver plays a crucial role in the biotransformation and elimination of chemicals, pollutants and drugs since the normal hepatocytes express a large panel of phase I, II and III metabolism enzymes [1] that catalyze the conversion of endogenous toxins and xenobiotics in hydrophilic products that are more easily eliminated in urine and bile. As a consequence, the liver that accumulates noxious compounds is also a main target of toxicity triggered by xenobiotics themselves and their down-stream reactive metabolites as demonstrated by the fact that a frequent cause for the market withdrawals of approved medications is the liver toxicity evidenced by the pharmacovigilance agencies after initial drug approval [2].

The establishment of metabolism pathways and the toxicity assessment of xenobiotics are technically difficult, often expensive and time-consuming processes that require the use of both in vitro cell systems [3] and in vivo animal models prior to any clinical trials in humans [4]. Among the different in vitro hepatic cell systems available, primary cultures of human hepatocytes are recognized as the most pertinent cell systems to accurately predict metabolism pathways and regulat phase I, II and III enzyme expressions and hepatotoxicity by synthetic compounds [1,4,5]. However, primary cultures of hepatocytes have several drawbacks considerably limiting their use. These cells are isolated from surgical biopsies of patients suffering from hepatic diseases, which lead to great variations in metabolic activities including enzymes involved in drug metabolism such as the cytochrome P450 enzymes (CYP). Furthermore, the reproducibility of the experiments is impaired by the fact that hepatocytes are isolated from different donors with variations in age, liver diseases and treatments [1,4]. In addition, the hepatocytes in primary cultures have a short lifespan. Thus, because of all these drawbacks as well as the relative shortage in human biopsies and the constraining ethical regulation, a limited number of laboratories have access to these culture models.

The hepatoma cell lines represent an attractive alternative to the use of human hepatocytes in primary culture to study the toxicity [3] and genotoxicity of xenobiotics [4,6]. These cell lines, however, express drug metabolism enzymes at low levels compared to normal hepatocytes in the liver and in primary cultures [1,7]. To circumvent this drawback, subclones of hepatoma cells have been derived such as HBG [8] and HepG2C3A that express higher amounts of at least some CYP [9]. Similarly, recombinant hepatoma cell lines have been generated through stable transfection of genes encoding drug metabolism enzymes [10] or liver-specific transcription factors driving transcription of liver-specific functions [11]. Most hepatoma cell lines show, however, reduced expression levels of phase I and II enzymes and limited induction upon treatments with their canonical xenobiotic inducers.

The human hepatoma HepaRG cells are bipotent hepatic progenitors actively proliferating at low density but are capable of differentiating into biliary- and hepatocyte-like cells when kept as a confluent cell monolayer in appropriate culture medium [12]. This cell model represents a unique in vitro cell system to investigate the molecular signalling pathways controlling the hepatocyte differentiation [13,14]. Moreover, HepaRG hepatocyte-like cells that express most of the major phase I, II and III enzymes [15,16,17] provide a valuable in vitro alternative model to primary cultures of hepatocytes and are used worldwide for studying the metabolism [15,18,19], drug toxicity [20,21] and genotoxicity of xenobiotics [4,22,23].

In the assessment of drug hepatotoxicity, an important aspect is the induction of drug metabolism enzyme expressions by xenobiotics in order to evidence the activation of metabolic pathways [4]. Beyond the study of the drug metabolisms, the expression of phase I and II in human hepatocytes is also strongly regulated by endogenous noxious compounds, hormonal factors such as growth factors and cytokines [24,25], and hypoxia [19]. In order to study the regulation of expression of drug metabolism genes, the mRNA and/or protein levels must be measured and, when an assay is available, the quantification of their enzymatic activity is performed. These experiments require relatively large amounts of cells and analytical measurements or molecular biology experiments that are labour intensive. To overcome these problems, rapid high-throughput cell-based assays that measure cell responses and gene induction are being developed to provide early warning on metabolic pathway activations and toxicity [4]. Among these assays, whole cell biosensors in HepaRG [21,26,27] and HepG2 [26,27,28,29] hepatoma cell lines have been developed in order to detect mitochondrial dysfunction, toxicity, metabolism and genotoxicity [4].

In the present study, we describe the development and validation of whole cell-based biosensor recombinant hepatoma HepaRG cells that were transduced with lentiviral particles carrying the partial gene promoters of CYP1A1/2, 2B6 and 3A4, driving the transcription of the reporter Green Fluorescent Protein (GFP). Characterization of these CYP1A1/2-, 2B6- and 3A4-GFP transgenic biosensor HepaRG cells demonstrated that GFP expression correlated with the expression levels of liver-specific functions. Furthermore, the treatment of biosensor HepaRG cells with prototypical inducers of CYP via xenobiotic responsive elements present in their promoters, benzo(a)pyrene and phenobarbital, induced the GFP expression levels. In addition, we demonstrated that differentiated CYP1A1/2, 2B6 and 3A4 biosensor HepaRG cells can be used to show selective hepatocyte toxicity following exposure to hepatotoxic compounds such as the mancozeb.

## 2. Materials and Methods

### 2.1. Reagents

Benzo(a)pyrene (B(a)P), thioacetamide, mancozed and insulin were purchased from Sigma-Aldrich (Saint Quentin Fallavier, France). Phenobarbital (PB) was provided by Sanofi-Aventis (Gardenal). William’s E medium was purchased from Eurobio (Les Ulis, France). Fetal calf serum (FCS) was obtained from Lonza (Levallois-Perret, France). Penicillin, streptomycin and L-glutamine were purchased by Life Technologies (Saint Aubin, France).

### 2.2. HepaRG Cell Culture

HepaRG cells were seeded at a density of 3 × 10^4^ cells/cm^2^. These cells were cultured in William’s E medium supplemented with 10% of FCS, 5 µg/mL insulin, 2 mM L-glutamine, 50 units/mL penicillin, 50 µg/mL streptomycin and 50 µM Hydrocortisone hemisuccinate sodium salt. Two weeks after plating, the cells were maintained for 2 more weeks in the same William’s E medium further supplemented with 2% dimethylsulfoxide (DMSO) in order to obtain the full hepatocyte differentiation [13,30]. For half maximal inhibitory concentration (IC_50_) assays, parental differentiated HepaRG cells were exposed to various concentrations of thioacetamide (1 to 250 mM) and mancozeb (2.5, 5 and 22.5 μM) for 24 h. For the induction of selective cytotoxicity, CYP-GFP biosensor HepaRG cells were treated with 3.5 μM of mancozeb for 24 h.

### 2.3. GFP Reporter Dene Constructs.

DNA fragments corresponding to the 5’ flanking region of Homo sapiens CYP1A1/CYP1A2 locus (bidirectional 5’ flank CYP1A1/2 promoter region) (−1679/+80 base pairs upstream of exon 1), CYP2B6 gene (−1995/+1 base pairs) and distal CYP3A4 gene enhancer region (−1429/+1 base pairs) were cloned into the lentiviral expression pLV-GFP vector upstream of the GFP reporter gene (Supplementary Material 1 for promoter sequences). The CYP3A4 promoters have been previously described [19,31,32].

### 2.4. Lentiviral Infection and Selection of Biosensor HepaRG Cells.

The CYP1A1/2, CYP2B6 and CYP3A4 promoter sequences were subcloned into the lentiviral expression pLV-GFP backbone upstream of the GFP reporter genes using the KpnI (5’ end) and SacI (3’ end) restriction sites. The production of lentiviral particles was performed in HEK293T packaging cell line (Vectalys, Toulouse, France). Proliferating progenitor HepaRG cells seeded in 24-well plates were infected for 48 h at a multiplicity of infection of 5, 24 h after plating the cells. After 2 days, the mediums were discarded and cell monolayers were washed with regular medium. Two weeks later, GFP positive hepatocyte-like HepaRG cells were sorted by flow cytometry. These GFP positive cells were expanded and frozen prior to further experiments.

### 2.5. Immunoblotting Blotting

For immunoblotting, culture medium was discarded and adherent cells were washed once with cold PBS and lyzed in the dish wells using lysis buffer: 50 mM HEPES pH 7.9, 150 mM, NaCl, 0.1 mM EDTA, 10% glycerol, 0.5% Tween 20 supplemented with protease inhibitors (EDTAfree, Roche). Following a brief sonication, total protein contents of cell extracts were quantified using Biorad protein reagent assay. For immunoblotting, 20 µg of protein was separated on NuPAGE^®^ Novex^®^ Bis-Tris 4–12% gels kit (Invitrogen) and transferred to PVDF membranes (Trans-blot^®^ Turbo™ Transfer System, Biorad) prior to immunoblotting using the following primary antibodies: CYP3A4 (anti-human CYP3A4 rabbit polyclonal antibody, AB1254, Millipore Sigma), CYP1A1/2 (anti-human CYP1A1/2 mouse monoclonal antibody, ABIN614141, Daiichi Pure Chemicals Co., Tokyo, Japan) and CYP2B6 (anti-human CYP2B6 rabbit polyclonal antibody, MBS9409762, Daiichi Pure Chemicals Co., Tokyo, Japan), CYP2E1 (anti-human CYP2B6 rabbit polyclonal antibody, PA26, Oxford Biomedical, MI, USA), GSTA1 and Mu (Biotrin, Dublin, Ireland), albumin and transferrin (Kent laboratories, WA, USA), HSC70 (B-6, sc-7298, Santa Cruz Biotechnology), GFP (mAb 8758, Abnova), and cyclin A (anti-human cyclin A rabbit polyclonal antibody, H-432, sc-751, Santa Cruz Biotechnology). Primary antibodies were detected using secondary goat or rabbit antibodies coupled to horseradish peroxidase (Dako, Denmark), and detection of the immune complex was performed by chemiluminescent detection (Pierce™ ECL Substrate). The signals were quantified with scanning densitometry using the Quantity One software program (Bio-Rad Laboratories, Hercules, CA, USA). Densitometry data were normalized with the loading control HSC70.

### 2.6. RNA Expression by RetroTranscription Quantitative Polymerase Chain Reaction

After discarding the culture medium and brief washing with cold PBS, cells were lysed in dish wells and the total RNA was extracted using a Macherey-Nagel NucleoSpin^®^ RNA kit. Following elution, quantification of total RNAs was performed by measuring the optical density at 260 nm. Then, reverse transcription was performed using 500 ng of total RNAs and High capacity cDNA reverse transcription kit (Applied Biosystems). The RNA expression levels of specific genes were measured with SYBR Green PCR Master Mix kit (Applied Biosystems) using the ABI PRISM 7900HT instrument. TATA Binding Protein (TBP) genes were used as normalizing genes. Primers sequences are shown in Table 1:

### 2.7. Fluorescence Microscopy and Flow Cytometry Analyses.

The cellular DNA was stained using the BD Cycletest™ Assay (BD Biosciences) and detected using a FACSCalibur™ equipment (BD Biosciences) using the CellQuest™ software. The DNA content was analyzed for 5000 cells. Percentages of cells in G0/G1, S and G2/M phases was established using the ModFit LT™ software (Supplementary Materials). The GFP was detected in HepaRG cells by fluorescence microscopy using Zeiss Inverted Microscope, and photographs were analyzed with the AxioVision Software. The quantification of GFP was carried out by flow cytometry: The cells were detached with trypsin and resuspended in William’s E medium containing FCS. The fluorescence intensity of 10,000 cells was analyzed with a Becton Dickinson le LSRFortessa™ X-20 (cytometry core facility of the Biology and Health Federative research structure Biosit, Rennes, France) to measure the fluorescence of the GFP positive cells. Flow cytometry data were analyzed using DIVA software (Becton Dikinson).

### 2.8. Statistical Analyses

The Results were expressed as mean ± SD (the standard deviation) of two or three independent experiments including three to six independent culture wells. Statistically significant variations after treatment were compared with controls using Student’s test with Excel software; * p value < 0.05, ** *p* < 0.01 and *** *p* < 0.001.

## 3. Results

### 3.1. Subsection

#### 3.1.1. Expression of Phase I and II Enzymes in HepaRG Hepatocyte-Like Cells.

**Figure 1:**
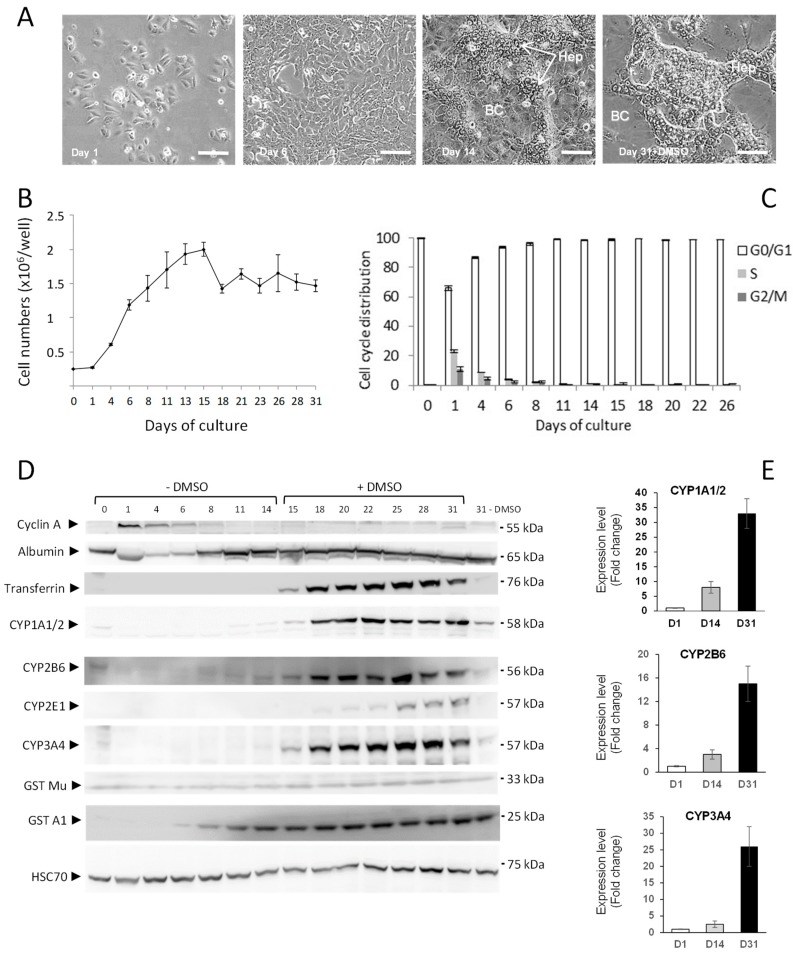
Proliferation and differentiation of HepaRG cells. Morphology in phase contrast of HepaRG cells at different stages of differentiation after plating (**A**). At day 1: bipotent progenitors at low density; 1 day after trypsination; subconfluent HepaRG cells at day 7 after trypsination, committed HepaRG hepatocyte-like (Hep) and biliary (BC) cells at day 14 post trypsination; and highly differentiated hepatocyte-like HepaRG and biliary cells 30 days after passage. To obtain full differentiation, cells were maintained for 2 weeks in culture medium supplement with 2% DMSO. Scale bar: 100 μm. Time course of cell counts of HepaRG cells at different times after cell plating (**B**). Percentages of cells in the different phases of the cell cycle (DNA content: G0/G1, S and G2/M) measured by flow cytometry at different times after cell plating (**C**). Immunoblotting of cyclin A, albumin, transferrin, CYP2B6, CYP2E1, CYP3A4, CYP1A1/2, GST Mu, GSTA1 and HSC70 used as a loading control, during the proliferation and differentiation of HepaRG cells (**D**). Densitometry analysis of CYP1A1/2, 2B6 and 3A4 immunoblottings at days 14 and 31 expressed in fold change compared to expression at day 1 and normalized with HSC70 protein levels (**E**).

The most appropriate procedure to expand HepaRG cells is to culture them over 2 weeks between two passages (Figure 1A). As previously reported [4,12,13,14], HepaRG cells actively proliferate during the first 8 to 10 days after seeding as confirmed by the increasing cell counts and the high numbers of cells in S and G2/M phases during this period (Figure 1B,C). Two weeks after cell seeding, the proliferation activity strongly decreased and over 95% of cells became quiescent (G0/G1 phase) while nearly 50% of quiescent HepaRG cells committed toward the hepatocyte-like cell lineage as demonstrated by the appearance of well-defined colonies of hepatocytes and the high expression of albumin detected by immunoblotting (Figure 1D).

To further enhance the expression of hepatocyte-specific functions, especially cytochrome P450’s, committed HepaRG are cultured for 2 more weeks with culture medium supplemented with 2% DMSO. Hepatocyte-like cells complete their differentiation and undergo drastic morphological changes to give rise to well-defined colonies of hepatocytes characterized by a dark cytoplasm, a large nucleus with a single nucleolus, and functional neo-canaliculi (Figure 1A), while the overall cell number is slightly reduced following DMSO treatment (Figure 1B). In most reports, the expression of liver-specific functions in HepaRG cells has been investigated at the mRNA levels [14] and/or by the quantification of drug metabolism enzyme’s catalytic activities [15]. In our study, we studied the expression of several phase I and II proteins by immunoblotting during a 31-day time-course to establish their sequential activation during the first 2 weeks of expansion and the 2 weeks of stimulation by DMSO treatment (Figure 1D). The proliferation was correlated with the expression of the cyclin A from day 1 to day 8 after cell seeding. Confluent cells detached by trypsin and seeded at low density (day 0) express high levels of albumin, confirming that HepaRG cells are committed to the hepatocyte lineage. The albumin expression was decreased during the active phase of proliferation between days 1 and 8 and increased until day 14. The addition of DMSO to the culture medium did not further enhance its expression. In contrast, transferrin, another plasma protein secreted by the hepatocytes, was barely detectable in proliferating and quiescent HepaRG cells during the first 2 weeks post-seeding. Its expression was induced by the DMSO treatment since at day 31, in absence of DMSO, the transferrin was expressed at very low levels. The GSTA1 and GST Mu were expressed in proliferating cells; their expression increased when cells became quiescent and the treatment by DMSO did not significantly increase their expression levels. In contrast, the expressions of the CYP1A1/2, CYP2E1, CYP3A4 and CYP2B6 had very low levels in quiescent cells at day 14, were undetectable during proliferation, and strongly increased in HepaRG hepatocyte-like cells upon stimulation by DMSO. Our data confirmed the commitment to the hepatocyte lineage following an active phase of proliferation, and full hepatocyte differentiation during the 2-week treatment by DMSO [4].

#### 3.1.2. Establishment of Biosensor HepaRG Cells

**Figure 2:**
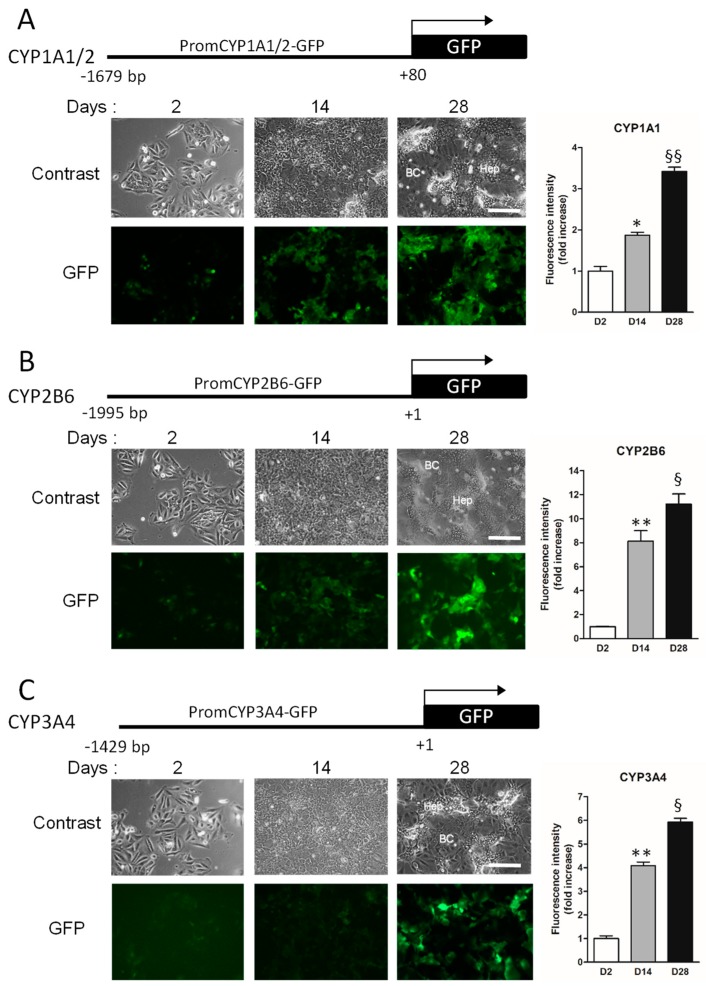
Schematic representation of CYP’s promoter constructs and establishment of biosensor HepaRG cell lines. CYP1A1/2 (**A**), CYP2B6 (**B**) and CYP3A4 (**C**) promoters were used in GFP reporter constructs. Visualization of the GFP by fluorescent microscopy and quantification of GFP fluorescent intensity by flow cytometry (charts) in the promCYP1A1/2-, CYP2B6- and CYP3A4-GFP HepaRG biosensor cells at day 2 after plating (progenitor HepaRG cells); at day 14 when confluent HepaRG cells have committed to biliary/cholangiocyte (BC) or hepatocyte (Hep) cell lineages; and at day 28 in differentiated HepaRG cells following 2 weeks of culture in DMSO-supplemented culture medium. Scale bar: 400 μm. Statistics: Fluorescence intensities were significantly higher in cells at day 14 (* *p* value < 0.05 and ** *p* < 0.01) versus cells at day 2, and in cells at day 28 (^§^ *p* value < 0.05 and ^§^ *p* < 0.01) versus cells at days 2 and 14.

In order to establish stable CYP1A1/2, CYP2B6 and CYP3A4 biosensor HepaRG cells, we subcloned the partial promoter sequences of the CYP1A1/2, CYP2B6 and CYP3A4 (Supplementary Materials) in the lentiviral pLV-GFP backbone expression vector (Figure 2). The CYP promoters were thus supposed to regulate the transcription of the GFP open reading frame and to allow the expression of GFP detected in situ by fluorescence microscopy and by flow cytometry to quantify the fluorescence intensities in HepaRG cells.

After lentiviral transduction and selection of recombinant cells stably transduced, we studied the expression of the GFP in these derived HepaRG cell lines in order to evaluate the patterns of transcription of the different CYP promoter-GFP transgenes during the proliferation and differentiation processes taking place over 30 days after cell splitting (protocol described in Figure 1). The GFP expression was detected in situ by fluorescent microscopy and quantified by flow cytometry (Figure 2). Visualization of the GFP by fluorescent microscopy demonstrated the low transcriptional activities of the CYP1A1/2, CYP2B6 and CYP3A4 promoters within the integrated lentiviral transgene at day 2 after HepaRG cell seeding. For all the transgenes, the GFP expression increased at days 14 and 28 after stimulation by DMSO and was much stronger in colonies of hepatocyte-like cells compared to the very low intensity of fluorescence detected in cholangiocyte-like cells.

**Figure 3:**
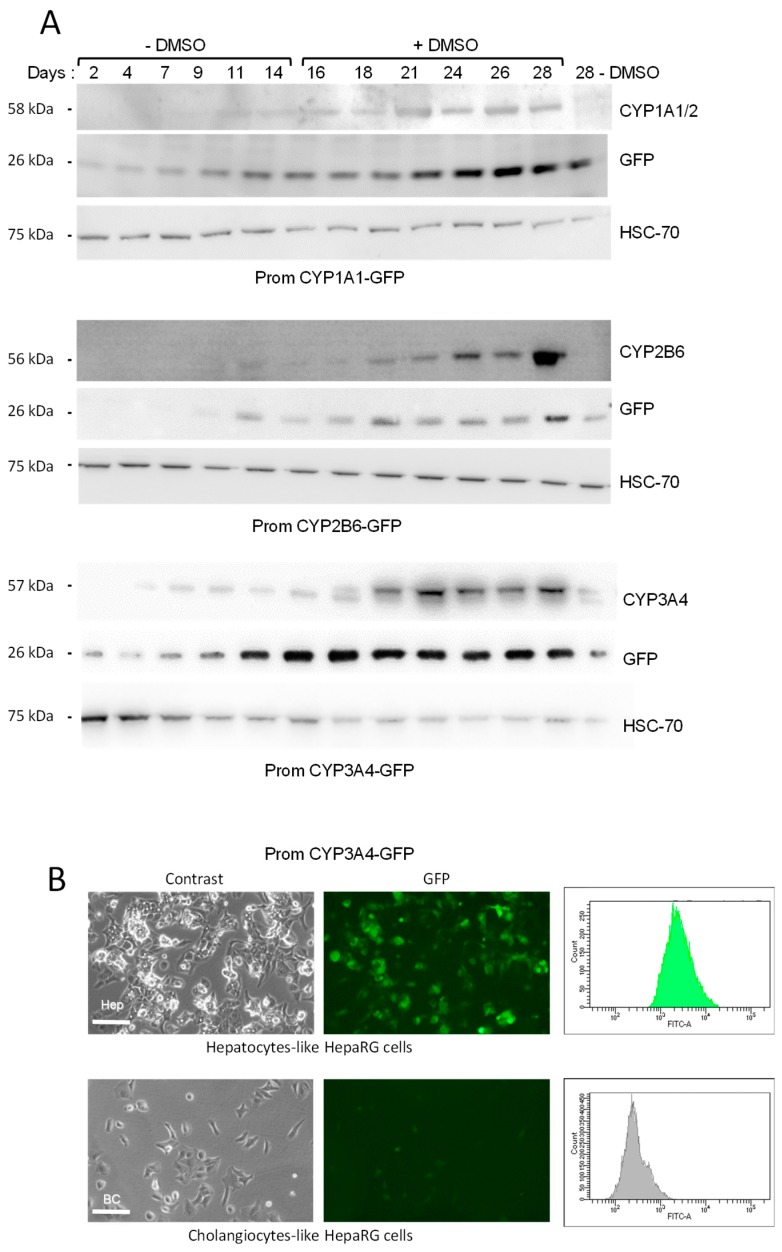
Correlated expression of GFP and endogenous CYP1A1, CYP2B6 and CYP3A4. Immunoblotting of endogenous CYP1A1, CYP2B6 or CYP3A4 and GFP in promCYP1A1-, CYP2B6- and CYP3A4-GFP HepaRG cells (**A**). HSC70 was used as loading control. Expression of GFP in hepatocytes-like and cholangiocytes-like HepaRG cells in enriched cell populations (**B**). Both cell types were selectively detached from differentiated CYP3A4-GFP biosensor HepaRG cells using a mild-trypsin detachment protocol previously described [13], and GFP expression was visualized by fluorescence microscopy and quantified by flow cytometry. Scale bar: 200 μm.

These data of in situ GFP visualization were confirmed by the GFP protein detection by Western blot (Figure 3A). A time-course analysis of the GFP expression in the CYP1A1/2-, CYP2B6- and CYP3A4-GFP HepaRG biosensor cells was performed and compared to the expression of the corresponding endogenous CYP proteins. Immunoblotting showed very similar expression patterns for the GFP and the corresponding endogenous CYP (Figure 3A), demonstrating that the endogenous CYP gene and the corresponding transgene containing the CYP promoter driving the GFP expression shared similar transcriptional regulation during the process of proliferation and differentiation in the HepaRG cells [4].

Cultures of differentiated HepaRG cells include both hepatocyte- and cholangiocyte-like HepaRG cells. To further demonstrate that GFP was mainly expressed in hepatocyte-like HepaRG cells and not in cholangiocytes, the two cell types were selectively detached using mild trypsinization as previously described [13], and fluorescence intensity was analyzed (Figure 3B). Enriched cultures of hepatocyte-like HepaRG cells showed high levels of fluorescence while the GFP expression was much lower in cholangiocyte-like cells.

#### 3.1.3. Induction of GFP Expression in Biosensor HepaRG Cells by Xenobiotics

**Figure 4:**
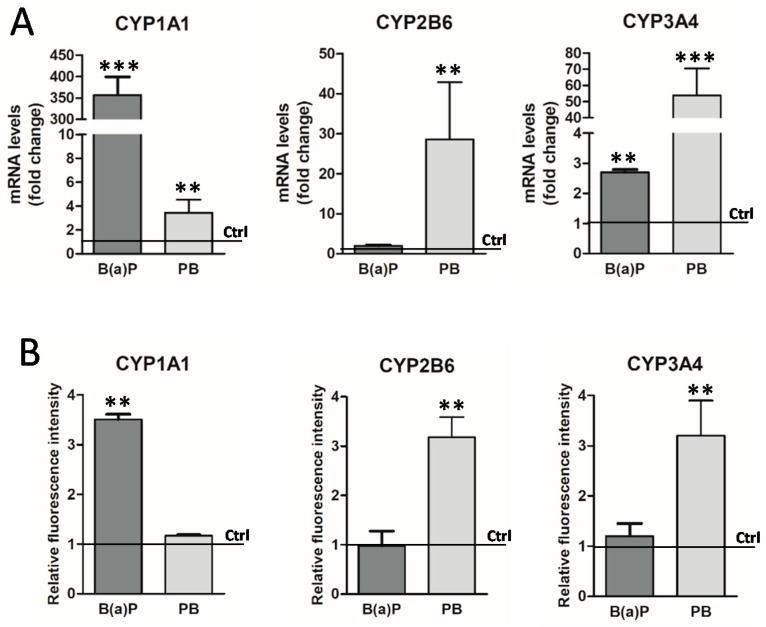
Induction of GFP expression in biosensor HepaRG cells by drugs. Expression levels of endogenous CYP1A1, CYP2B6 and CYP3A4 mRNAs in biosensor HepaRG cells (**A**). HepaRG cells were treated for 24 h with prototypical inducers of CYP genes, the phenobarbital (PB) and Benzo(a)pyrene (B(a)P). The endogenous CYP1A1, CYP2B6 and CYP3A4 RNA expression levels were studied by quantitative PCR and results were expressed as fold changes compared to untreated control cells. Quantification of the GFP expression by flow cytometry in promCYP1A1/2, CYP2B6 and CYP3A4-GFP biosensor HepaRG cells following treatments with B(a)P and PB (**B**). Results were expressed as fold changes in fluorescence relative to the signal measured in untreated control cells (Ctrl) arbitrarily set as 1. Statistics: * *p* value < 0.005, ** *p* < 0.01, *** *p* < 0.001.

We next compared the expression of endogenous CYP genes at the mRNA levels and the GFP expression by flow cytometry following treatment with prototypical inducers of CYP450 genes in differentiated biosensor HepaRG cells (Figure 4). Differentiated biosensor HepaRG cells were treated for 48 h with benzo(a)pyrene B(a)P at 5 μM and phenobarbital (PB) at 1.5 mM, then, the expression of endogenous CYP1A1, 2B6 and 3A4 mRNA was analyzed by RT-qPCR (Figure 4A). The CYP1A1/2 mRNA levels were strongly and significantly induced (~350-fold) by B(a)P and to a much lesser extent by PB (~3.7-fold). The expression of CYP2B6 and CYP3A4 showed a 30- and 50-fold increase after treatment by PB, respectively, while B(a)P led to a 2.7 increase in CYP3A4 expression and no effect on CYP2B6 mRNA levels [4].

We next evaluated the induction of GFP expression by flow cytometry in the same biosensor HepaRG cells following treatments with B(a)P and PB (Figure 4B). Despite much weaker inductions (three- to four-fold) in GFP expression compared to the strong inductions of endogenous CYP mRNAs (Figure 4A), we showed that CYP1A1/2-GFP reporter transgene was also significantly induced by B(a)P with a three- to four-fold increase in fluorescence intensities in biosensor hepatocyte-like HepaRG cells while the fluorescence was not significantly induced by PB. Conversely, the CYP2B6 and CYP3A4-GFP reporter transgenes were significantly induced by PB with three to four-fold increases in fluorescence intensities but not by B(a)P.

#### 3.1.4. Detection of Selective Hepatocyte-Like Cytotoxicity

A main feature of the progenitor HepaRG cells is their ability to differentiate into two hepatic lineages leading to a differentiated coculture model of hepatocyte- and cholangiocyte-like cells (Figure 1). Thus, we next evaluated whether the CYP-GFP biosensor HepaRG cells could be used to evidence selective cell death of hepatocyte-like HepaRG cells triggered by drugs known to induce hepatocyte toxicity. We selected two hepatotoxic fungicides, the thioacetamide-inducing centrolobular hepatocyte necrosis in vivo [33,34,35] and the mancozed triggering necrosis/apoptosis in rat liver [36] and in HepG2 hepatoma cells [37].

**Figure 5:**
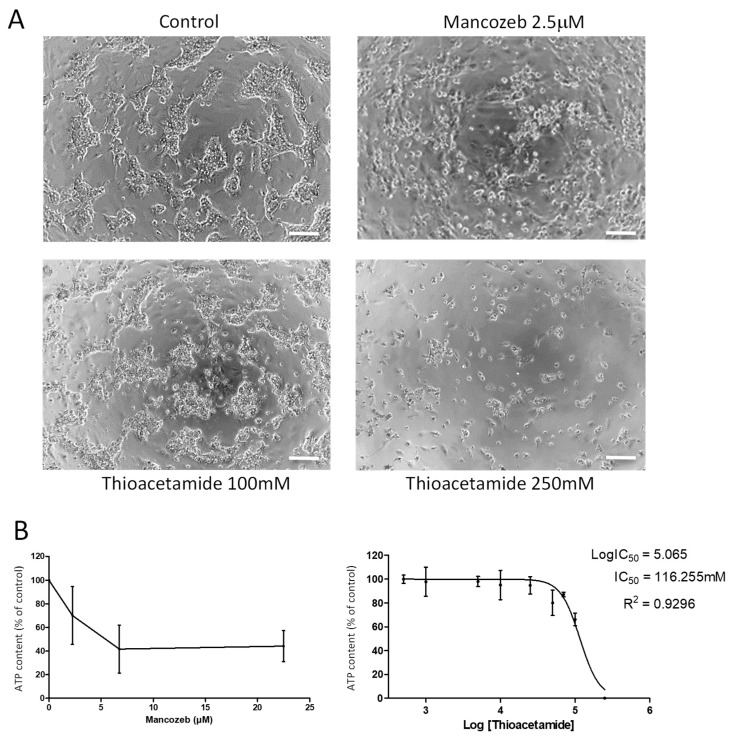
Viability of differentiated HepaRG cells upon treatment by thioacetamide and mancozeb. Viability of HepaRG cells following a 24 h exposure to various concentrations of thioacetamide (1 to 250 mM) and mancozeb (2.5, 5 and 22.5 μM) was evaluated by phase contrast microscopy (**A**) and by measuring the relative ATP content compared to untreated control cultures (**B**).

In order to determine whether these two compounds induce the cell death of differentiated HepaRG cells, the morphological alterations were monitored and a cell viability was assayed by measuring the ATP content following treatments by increasing the concentrations of thioacetamide and mancozed (Figure 5). Twenty-four hours after exposure to 2.5 μM or higher concentrations of mancozeb, colonies of hepatocyte-like HepaRG cells were disrupted with cells that rounded up while cholangiocyte-like cells remained adherent (Figure 5A). This observation suggested a selective hepatocyte toxicity induced by the mancozeb. This conclusion was strengthened by the dose-dependent decrease in ATP content at 2.5 and 7 μM with a half maximal inhibitory concentration (IC_50_) at 5 μM ± 0.5 (Figure 5B). The highest dose of 22.5 μM did not further reduce the ATP content, confirming that a large fraction of the HepaRG cell population was resistant to very high doses of mancozed. In contrast, the thioacetamide induced a dose-dependent decrease in ATP content leading to complete cell death of all the HepaRG cells for concentrations higher than 100 mM, demonstrating that thioacetamide triggers cell death of both cholangiocytes- and hepatocyte-like HepaRG cells (Figure 5A).

**Figure 6:**
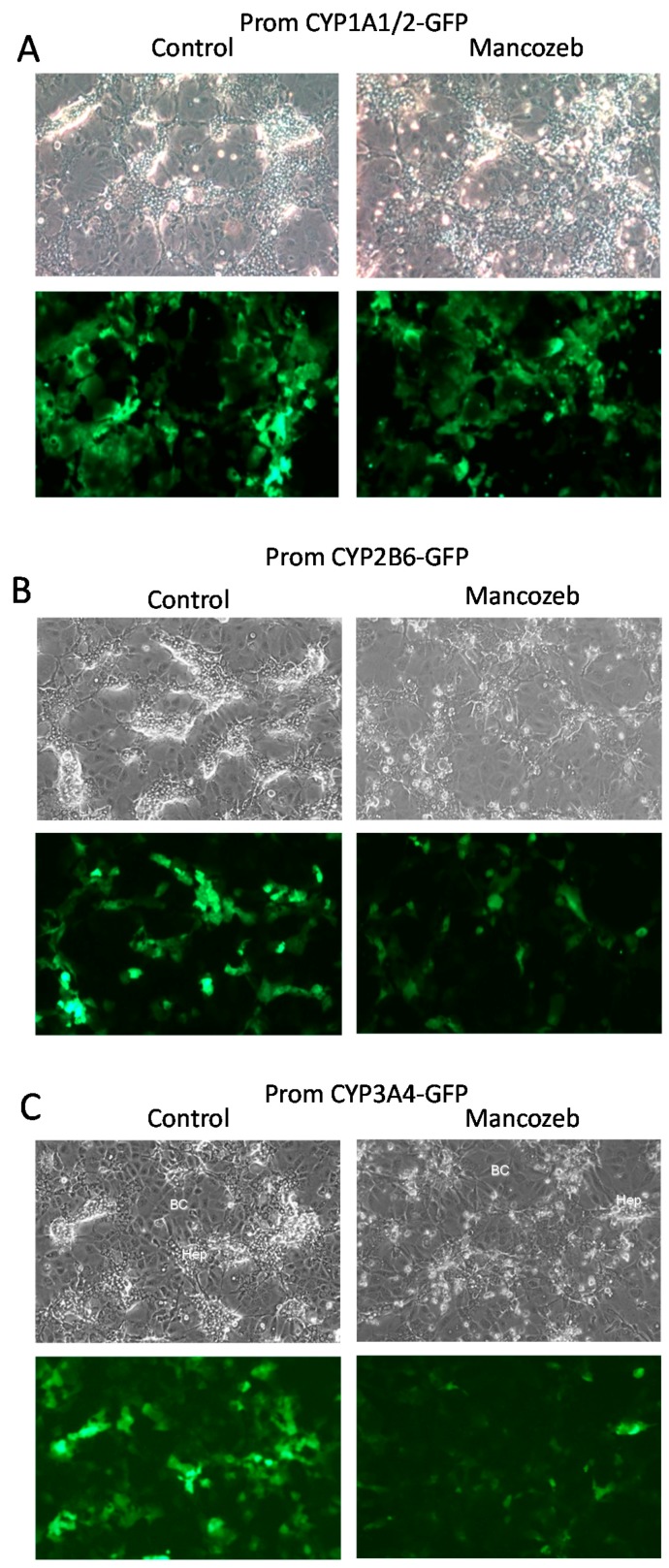
Selective toxicity of the mancozeb towards CYP-GFP biosensor hepatocyte-like HepaRG cells. Phase contrast microscopy (upper panels) and detection of GFP by fluorescence microscopy (lower panels) in promCYP1A1/2 (**A**), promCYP2B6 (**B**) and promCYP3A4 (**C**) biosensor HepaRG cells exposed to mancozed for 24 h. Scale bar: 400 μm. BC: biliary- or cholangiocytes-like cells, Hep: hepatocyte-like HepaRG cells.

**Figure 7:**
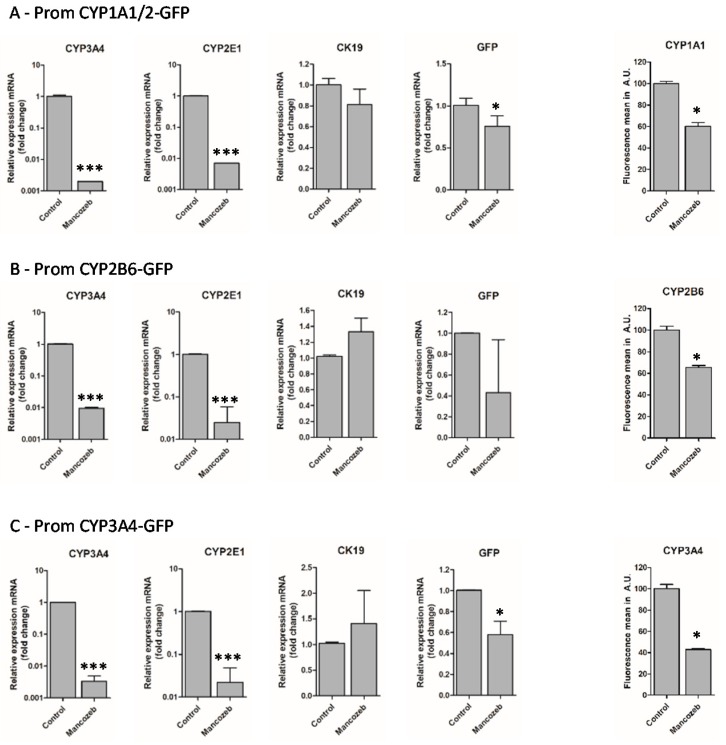
Selective toxicity of toxic compounds towards hepatocyte-like HepaRG cells. The mRNA expression levels of endogenous CYP3A4, CYP2E1, cytokeratin 19 (CK19) and GFP were evaluated by RT-qPCR, and the GFP fluorescence intensities were measured by flow cytometry in promCYP1A1/2- (**A**), promCYP2B6- (**B**) and promCYP3A4 (**C**) biosensor HepaRG cells either untreated (control cells) or exposed to mancozed for 24 h. The mRNA expression levels in control cells were arbitrarily set as 1 while the mean of fluorescence in control cells was set as 100%. Statistics: * *p* value < 0.005, *** *p* < 0.001.

To further evaluate whether the promCYP1A1/2-, CYP2B6- and CYP3A4-GFP biosensor HepaRG cells could be used to detect selective hepatocyte cytotoxicity, differentiated HepaRG cell cultures were then treated with mancozeb for 24 h and the GFP expression was visualized by fluorescence microscopy (Figure 6) and quantified by flow cytometry (Figure 7). As previously observed (Figure 5), the mancozeb induced a strong alteration of hepatocyte-like HepaRG cell colonies visible in phase contrast and fluorescence microscopy with a reduction of the GFP expression in promCYP1A1/2-, promCYP2B6- and promCYP3A4-GFP biosensor cells (Figure 6A–C and Figure 7A–C). Quantification by flow cytometry showed a 40 to 50% decrease in GFP mRNA expression levels and fluorescence intensity in mancozeb-treated GFP biosensor cells (Figure 7). The decrease in GFP expression also correlated with the strong reduction in the mRNA expression levels of endogenous hepatocyte-specific genes CYP3A4 and CYP2E1 following exposure to mancozed in the GFP-biosensor HepaRG cells. In contrast, the mRNA expression levels of the cytokeratin 19, which are expressed at higher levels in cholangiocytes- than in hepatocyte-like HepaRG cells [13], did not decrease upon treatment by mancozeb (Figure 7).

These data indicated that mancozeb induced cell death preferentially in promCYP1A1/2-, promCYP2B6- and promCYP3A4-GFP biosensor hepatocyte-like HepaRG cells while cholangiocyte-like cells showed no major morphological changes.

## 4. Discussion

The most relevant feature of the hepatocyte-like HepaRG cells is their remarkable levels of expression of most specific liver functions since nearly 90% of the genes found expressed in primary culture of human hepatocytes are also expressed in differentiated HepaRG cells [38]. In addition, these cells exhibit a high metabolism capacity with very active mitochondrial oxidative phosphorylation [39] and xenobiotic metabolism [22,40,41]. This unique cell model has been used to develop whole cell biosensors [21,26,27] for studying gene inductions activation of the hepatic metabolic pathway and cytotoxicity. We have also recently developed an original transfection procedure to efficiently introduced exogenous DNA in hepatocyte-like HepaRG cells [42]. This protocol was used to transfect in differentiated HepaRG-like hepatocytes a biosensor plasmid expressing the luciferase reporter gene under the transcriptional control of a consensus Xenobiotic Responsive Element motif and a minimal promoter. This transgene transiently expressed was able to sensor the induction of the Xenobiotic Responsive Element (XRE) following treatments by the drug-activating transcription factor, binding to the consensus xenobiotic responsive element sequence through the detection of luciferase activities [42].

This initial study prompted us to develop new biosensor HepaRG cell lines. Because of the raising interest in hepatocyte-like HepaRG cells for drug metabolism studies [40,41] and for analyzing the process of differentiation from hepatic progenitors towards hepatocytes, we herein report the establishment of three recombinant HepaRG cell lines that express the GFP under the transcriptional control of partial promoters of CYP1A1/2, CYP2B6 and CYP3A4 genes. We demonstrated that the CYP-GFP transgenes and the corresponding endogenous CYP genes shared similar expression patterns during proliferation and differentiation of HepaRG cells. As expected, differentiated hepatocyte-like HepaRG cells expressed GFP at higher levels compared to cholangiocyte-like cells. The prototypical drug-inducers benzo(a)pyrene and phenobarbital trigger the induction of GFP expression in prom-CYP biosensor HepaRG cells, which, however, remained in much lower magnitudes compared to the strong increases in mRNA levels of corresponding endogenous CYP genes detected by RT-qPCR. We and others have previously reported similar levels of inductions (3- to 10-fold) of such reporter genes [26,27,42]. These limited induction rates can be explained by the use of partial promoter gene sequences that most likely lack some of the regulatory elements such as distant enhancer sequences. In addition, the loci of the transgene insertions may also play an important role in the transcriptional regulation of the reporter gene. Furthermore, the CYP1A1/2-, CYP2B6- and CYP3A4-GFP biosensor HepaRG lines established in our study are pools of GFP positive cells. Individual clones could be further derived to isolate cells with higher rates of induction by drugs. Nevertheless, the pattern of GFP expression during differentiation of HepaRG cells correlated well with the expression of endogenous CYP proteins, and the induction by drugs observed in CYP1A1-, CYP2B6- and CYP3A4-GFP biosensor HepaRG cells was reproducible.

We also used the differentiated CYP-GFP biosensor HepaRG cells to evidence that the mancozeb triggered selective cytotoxicity of hepatocyte-like cells while cholangiocyte-like cells were not strongly affected by the treatment. In contrast, the thioacetamide induced a strong cytotoxicity towards both hepatocyte- and cholangiocyte-like HepaRG cells as previously reported in vivo and in vitro in primary cultures of rat hepatocytes [34,35].

These HepaRG cell-based biosensor systems present two major advantages. The CYP-GFP biosensor transgenes are integrated in the genome of HepaRG cells, leading to stable GFP expressions in these metabolically competent hepatoma cells that allow for the detection of the indirect-acting compounds on CYP expression without the use of exogenous metabolic activation. In addition, the variations in GFP fluorescence intensities of the GFP can be performed either by direct in situ fluorimetry quantification in culture microplates or by flow cytometry, which provide in both cases easy handling and data acquisition.

Together, our data demonstrated that the novel transgenic biosensor HepaRG cells described in this manuscript could be pertinent cell models for potential applications in the evaluation of xenobiotic safety and the assessment of drug hepatotoxicity. Further experiments are currently in progress to assess the inductibility of the CYP-GFP biosensor cells upon stimulation by other canonical inducers and to study the selective cytotoxicity of a larger collection of pesticides and environmental contaminants that were shown to induce genotoxicity in HepaRG cells [23].

## Figures and Tables

**Table 1:** Forward and reverse primers used for quantitative PCR.

	Forward	Reverse
CYP1A1	TCTTCCTTCGTCCCCTTCAC	ACACCTTGTCGATAGCACCA
CYP2B6	TTCCTACTGCTTCCGTCTATC	GTGCAGAATCCCACAGCTCA
CYP2E1	TTGAAGCCTCTCGTTGACCC	CGTGGTGGGATACAGCCA
CYP3A4	CTTCATCCAATGGACTGCATAAA	TCCCAAGTATAACACTCTACACACACA
CK19	TTTGAGACGGAACAGGCTCT	AATCCACCTCCACACTGACC
GFP	ACAACAGCCACAACGTCTAT	GGGTGTTCTGCTGGTAGTG
TBP	GAGCTGTGATGTGAAGTTTCC	TCTGGGTTTGATCATTCTGTA

## Supplementary Materials

The following are available online at https://www.mdpi.com/1424-8220/19/10/2245/s1, Figure S1.
