# Supplementary material for: Cytochrome P450 1A1/2, 2B6 and 3A4 HepaRG Cell-Based Biosensors to Monitor Hepatocyte Differentiation, Drug Metabolism and Toxicity
Source: Sensors (Basel). 2019 May 15;19(10):2245. doi: 10.3390/s19102245 (PMC6567340; doi:10.3390/s19102245)
Supplement: Supplementary file 1 [file sensors-19-02245-s001.pdf]

Supporting information

Cell cycle DNA content Analysis:
The DNA content was measured following propidium iodide staining using the BD Cycletest™ Assay (BD Biosciences) and detected using a FACSCalibur™ equipment (BD Biosciences). The gating of the cells was performed using dot plots of FL2-Width versus FL2-Area to gate the single cells. Then, cell cycle distribution was calculated with the FL2-A fluorescence intensity histograms obtained from the dot plots (FL2-W vs FL2-A).
The following documents show the dot plots and histograms for the time points at day 1, 6 and 14 after cell seeding, which were used to obtain the histogram presented in Figure 1C.

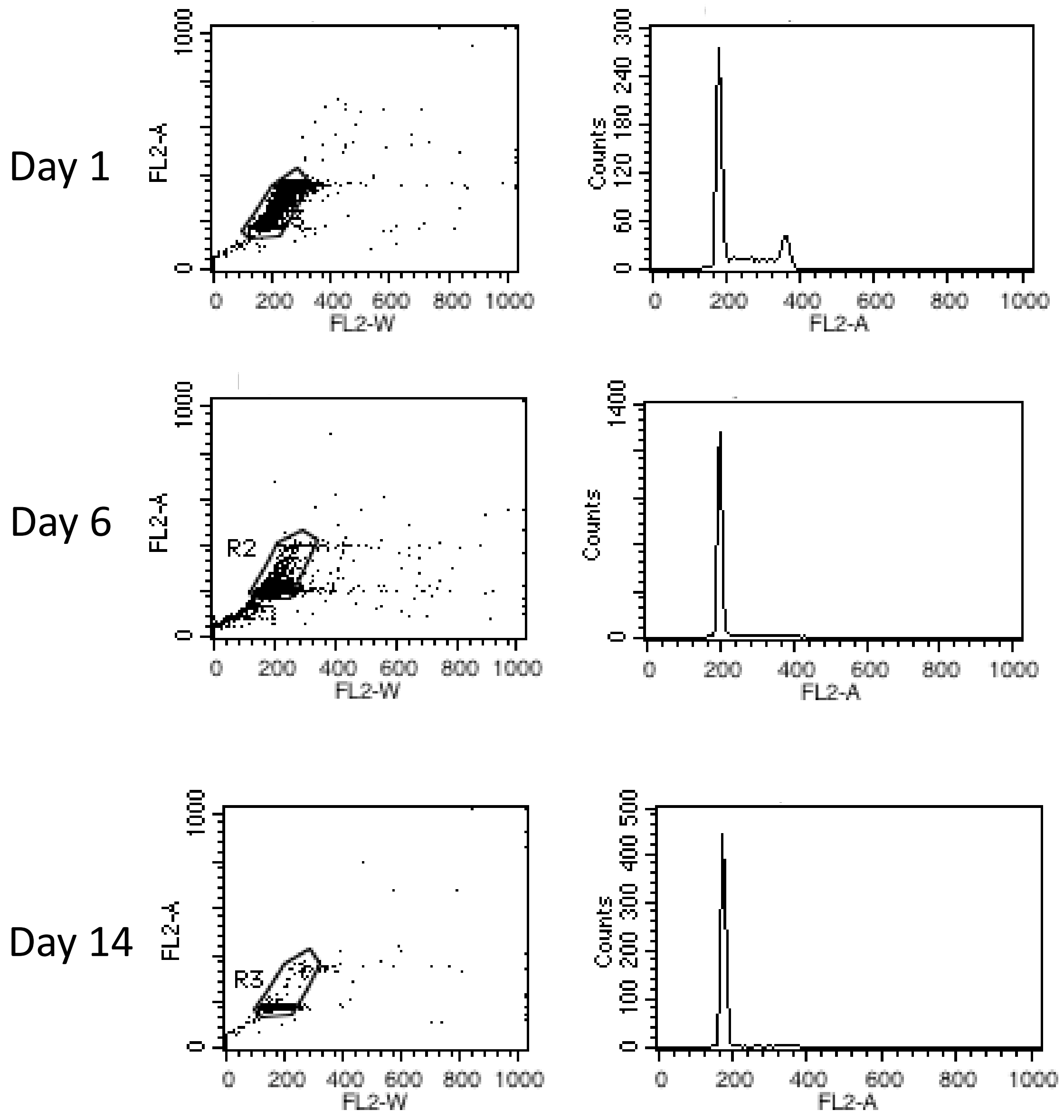

Supporting information 2

DNA sequences of the promoter regions subcloned into the lentiviral expression pLV-GFP backbone upstream of the GFP reporter genes using the KpnI (5’ end) and SacI (3’ end) restriction sites. Accession numbers of the 3 DNA sequences are indicated.

CYP1A1/2

```
LOCUS NG_008431 of 41009 bp, DNA linear, 20-SEP-2018
Homo sapiens CYP1A1_CYP1A2 locus, having a bidirectional 5' flank promoter
of 23,306 bp (CYP1A1-CYP1A2) on chromosome 15.
```

```
GGGCCCCCCCTCGAGGTCGACGGTATCGATAAGCTTCTGGCACATAGTAGTTGCTTGGTAAA
TATTTGTGCAATGCACGAGGCCGCATATGACCGGAATGGGAGGTGAGGGGATTATTTTCTGG
CCTGGACCAGCGACGGATGGAGGTGCCACCGGGTTGGGGAGCACGTCGGGGATGGCGCGTAA
CGATGTTAGCTGGGGCCAGGTTGAGCTAGGCACGCAAATACAACTTTTTTTTTCCTGGAAAC
CCTGTAACAGGAAGGTTCCGGAGGGCGGGACAGCGTCGGAGGCAGGCAGCTAGGCCATGCCA
AATGGCACTGGGGCTTCGTGTCGTGCCACAGGCGTGGACCGAAAATGCGGACACATGCAGGC
TGCCTCTCCTCGCAGGCAGAAGCCACACGCAGACCTAGACCCTTTGCACCGCATCCCCTTAT
TCAATCGCGCACCCGCCACCCTTCGACAGTTCCTCTCCCTCCACCCCAACCCCACGCCGCGC
GCGAGGCTGGCCCTTTAAGAGCCCCGCCCCGACTCCCTCCCCCCTCGCGTGACTGCGAGCCC
CCGCGCCGGGCCGGGGAATGGGTCGGCTGGGTGGCTGCGCGGGCCTCCGGTCCTTCTCACGC
AACGCCTGGGCACCGCGCCTCCGGGCCAGGTGGGGCGGGGACGGGCCGCCTGACCTCTGCCC
CCTAGAGGGATGTCGCCGGCGCACGCAAGCTAGCCGGGGGTAGGGTGGGGGCTCCGCGCCAG
GTGCCCCCTCCGTGGTCCCTGGGCCCGAGTCTTTCCGTGGCCCCCCGCCGCCGGATTTCTGT
GCTCTGCCAATCAAAGCACTAGCCACCCCGGGAGCCAAGAGGGACCCTCAAGGGCCGGTGGG
TCCTGGCTGGAGGGACCGCGCGTTGCAATCAGCACTAAGGCGATCCTAGAGGCTGCGAGGAG
CCGCTAGTGAGCGCTCAGCGAGCCTGCCCCTTCGCCATCCATTCCGATCCTTCAATCAAGAG
GCGCGAACCTCAGCTAGTCGCCCGGGCTCTGGGGGACAGGTCCAGCCCCGCGGCGCCTCTGG
CCTTCCGGCCCCCGTGACCTCAGGGCTGGGGTCGCAGCGCTTCTCACGCGAGCCGGGACTCA
GTAACCCCGGGAAGAAGGTCACCACGGGGCAGCCCCGCCCCCGCCTGCCGAGTCCTGGTAGG
CTGTAGCGCTGGGGAGGCATCTGCACGCCCAGCGTTCCAGTGGGTGCAAAAATGACGAAGAG
GAGTCCCCGCGCCCCAGGATGGAGCTTCCCGTACCCTCTCTTCGGGCTGTCCTGGGACTTCT
CCCTCAAGCCCCCTCCTCGGCTGGGTTCTGCACTGCCCTTGGGACGCCTTGGAATTGGGACT
TCCAGGTGTTCCCAGCCCTCACCCCTCTATGTACAGGCACCGAGATGTGTCCCATAGTGGGT
TCTTGCCCACCCGACCCCCCACCCCCGCCGCCCTCCGCCACCTTTCTCTCCAATCCCAGAGA
GACCAGCCCGGTTCAGGCTGCTTCTCCCTCCATCTCAGCTCGCTCCAGGGAAGGAGGCGTGG
CCACACGTACAAGCCCGCCTATAAAGGTGGCAGTGCCTTCACCCTCACCCTGAAGGTGACAG
TTCCTTGGAACCTTCCCTGATCCTTGTGATCCCAGGCTCCAAGAGTCCACCCTTCCCAGAT
```

CYP2B6

LOCUS NG_055439 of 2469 bp DNA linear, 05-AUG-2017
Homo sapiens CYP2B6 promoter-proximal regulatory region (LOC110599575) on chromosome 19.

```
TTGGACAATGTAGCCCCAACCCACTGTATGACCTTGGGTAAGAAAAACAAACTGTCATTGCT
TCAATATCTCCATCTATAAAATGGGGATGGCAACAATACCTCACTAAGAGTGTGAAGACTGA
GTTACTGTGTGTAAAGCACTTCACGCCTCCCCATCGGTGCTTCACCCTGGGGCTGCAATGAG
CACCCAATCTTAGTGTCAGATGACACAGCACAGCAAGACCGAGGCCCTTGGTTCAGGAAAGT
CCATGCTGCCACCTCTTCAGGGTCAGGAAAGTACAGTTTCCACCTCTTACAAATAGGACTGT
TTGTCTGCTCCTCCTGGGTCAAAGTAACTTCGGGTTCAGGTCCTGGATCCAGCAAAGGGTTT
GCTTAACATTGCAAGAAAGATGTTGCCTCATGGTCAAAAGTCAGGCGTAGGATGAGACAGGC
AGACACGCACACATTCACACCCACGTTTTGCAAAGATGGACTGACCCTGTCAGAGGATGTGT
GGGTGAAGGTGCACAGTGAGGATAGAGACATATGGGAGTCCAGTAGACATCAATCAAACTGG
ACTCAGTTTGCACACACCTGGAGCTCAAGAGTCTCCAGGGGGAAAACAGAGACACAAAGTCA
GACAGAGAGAGAGCCAGAGAAATTTCCTGCACCGTGAAGATAGTCAGAGGCAGGGAAGAAAC
TCCTTAGCACTAGTTAGAGTGATCAGAAACCAAGAGGACCTGATCGCTGTACCTGCCAGGTC
TCAGTTTCTGTCTCCTTCCAACTGACCACCTCTTCCTCTGAGACTCACCAGTTCTGCATCTC
TTGCTCCTCCTTCTGTTTCTCCGACCACTTCCACCTGTGGCTGTCACAGAAGGGCGGATGAA
GGAGGGGACACTGGAGATAGACTCAGCATCTGCAGGCTTCCAAAGAGAGGGGCTAGGAGATC
CACCAACACACCAGCACAAATACACCAGCACACACAGATACACACAATTGGTTCATGTATTG
CTAGGTTACAGTTTGCTATGCTACAAAGGCAGTAGGCCAAATTTGATTGAATTGAATAATTC
CTTATTTTCATCAGCTTCTCCTTTTTTTTTTTTTTTTTTTTGAGATGGAGTATTGCTGTG
TCACCCAGGCTGGATTTTGAGATGGAGTATTGCTGTGTCACCCAGGCTGGAGTGCAGTGGTG
TAATCTTGGCTCACTGCAGCCTCCACCTCCCAGGTTCAAGTGATTCTCTTGCCTCAGCCTCA
CGAGTAGCTGGGATTAAAAGTACCCACCATCACGCCCGGCTAATTTTTGTGTTTTTAGTACA
GATGGGGTTTTGCCATGTGGGCCAGGATGGTCTCGAACTCTTGACCTCAATTGATCTGCCCC
CCTCAGCTTCCCAACGTGCTGGGATTACAGGTGTGAGCCACCGCACCCAGCCAGCCTCTCAG
TTTTGAACATGCACTACCACCACCTCCACAACACACAAATGTAAATGCACTTTCGTATATAA
AACTGTATAAATACGAGGAAGCTCATACACATGCAAGGATACACACATAAGCACCCCCAGAT
TCAACCACAGAAATATACGCCAGTACATTTGCATAAATTCAAACACCCCTTTACATGTAAAA
ATCATATAAGCACATACAGGGATGCAAGCAGGCATGGACAAATGCATGCAAGCACAGACAAA
CAGACAAAGCTAAGTAAAAAAGTGCAAGCTCACCTATGCTTACAAAAATAGACATACATATA
CCCACAAACCCACACACCCACACATTCACTTGCTCACCTGGACTTTGATAACTCTACCACTG
TATCCCTGCCAATATCTACAGAGTGGGTAAAGGGATAGGCATCAGGTCACTGGGTTGCCCAA
GCAGGAAGTCTGGGTTCCCTAACAACTTTTTCTAAGCTAATGCTCCTGGATGATGATGAAAA
AGGAGGTGGGGAATGGATGAAGTTTTATAACAGGGTGCAGAGGCAGGGTCAGGATAATATGC
CCAATTGGAGG
```

CYP3A4

LOCUS NG_054901 of 12769 bp DNA linear, 14-JUL-2017
Homo sapiens CYP3A4 5' regulatory region (LOC110366354) on chromosome 7.

AGTGATTCTGAGCCATGTCTATTTCTCCTGACTTTTCCTTGAAGCCCACGCTTGCTGATATT
TACAGCCGTGGTCATCACACTTACTTTTGTTCCTTTCTTCCTTCCTCCAACTCTGCATTAAA
TTCCAGGAACTTGCTTTCTGTGAAGTCTAGTCGAAGCTTGCATGCCTGCAGGTCGACCAGCT
CCTGGGGCCTGCCCTCCTCCCATTAGAAAATCCTCCACTTGTCAAAAAGGAAGCCATTTGCT
TTGAACTCCAATTCCATCCCCAAGAGGCTGGGACCATCTTATTGGAGTCCTTGATGCTGTGT
GACCTGCAGAGACCACTGCCCCATCATTGCTGGCTGAGGTGGTTGGGGTCCATCTGGCTATC
TGGGCAGCTGTTCTCTTCTCTCCTTTCTCTCCTGTTTCCAGACATGCAGTATTTCCAGAGAG
AAGGGGCCACTCTTTGGCAAAGAACCTGTCTAACTTGCTATCTATGGCAGGACCTTTGAAGG
GTTCACAGGAAGCAGCACAAATTGATACTATTCCACCAAGCCATCAGCTCCATCTCATCCAT
GCCCTGTCTCTCCTTTAGGGGTCCCCTTGCCAACAGAATCACAGAGGACCAGCCTGAAAGTG
CAGAGACAGCAGCTGAGGCACAGCCAAGAGCTCTGGCTGTATTAATGACCTAAGAAGATGGA
GTAGTCACCAGAAAGTCAGAAGGGATGACATGCAGAGGCCCAGCAATCTCAGCTAAGTCAAC
TCCACCAGCCTTTCTAGTTGCCCACTGTGTGTACAGCACCCTGGTAGGGACCAGAGCCATGA
CAGGGAATAAGACTAGACTATGCCCTTGAGGAGCTCACCTCTGTTCAGGGAAACAGGCGTGG
AAACACAATGGTGGTGAAGAGGAAAGAGGACAATAGGATTGCATGAAGGGGATGGAAAGTGC
CCAGGGGAGGAAATGGTTACATCTGTGTGAGGAGTTTGGTGAGGAAAGACTCTAAGAGAAGG
CTCTGTCTGTCTGGGTTTGGAAGGATGTGTAGGAGTCTTCTAGGGGGCACAGGCACACTCCA
GGCATAGGTAAAGATCTGTAGGTGTGGCTTGTTGGGATGAATTTCAAGTATTTTGGAATGAG
GACAGCCATAGAGACAAGGGCAGGAGAGAGGCGATTTAATAGATTTTATGCCAATGGCTCCA
CTTGAGTTTCTGATAAGAACCCAGAACCCTTGGACTCCCCAGTAACATTGATTGAGTTGTTT
ATGATACCTCATAGAATATGAACTCAAAGGAGGTCAGTGAGTGGTGTGTGTGTGATTCTTTG
CCAACTTCCAAGGTGGAGAAGCCTCTTCCAACTGCAGGCAGAGCACAGGTGGCCCTGCTACT
GGCTGCAGGTCCAGCCCTGCCTCCTTCTCTAGCATATAAACAATCCAACAGCCTCACTGAAT
CAC
